# A systematic review of early evidence on generative AI for drafting responses to patient messages

**DOI:** 10.1038/s44401-025-00032-5

**Published:** 2025-07-23

**Authors:** Di Hu, Yawen Guo, Yiliang Zhou, Lidia Flores, Kai Zheng

**Affiliations:** Department of Informatics, Donald Bren School of Information and Computer Sciences, University of California, Irvine, Irvine, CA USA

**Keywords:** Information systems and information technology, Health care

## Abstract

This systematic review synthesizes currently available empirical evidence on generative artificial intelligence (GenAI) tools for drafting responses to patient messages. Across a total of 23 studies identified, GenAI was found to produce empathetic replies with quality comparable to that of responses drafted by human experts, demonstrating its potential to facilitate patient–provider communication and alleviate clinician burnout. Challenges include inconsistent performance, risks to patient safety, and ethical concerns around transparency and oversight. Additionally, utilization of the technology remains limited in real-world settings, and existing evaluation efforts vary greatly in study design and methodological rigor. As this field evolves, there is a critical need to establish robust and standardized evaluation frameworks, develop practical guidelines for disclosure and accountability, and meaningfully engage clinicians, patients, and other stakeholders. This review may provide timely insights into informing future research of GenAI and guiding the responsible integration of this technology into day-to-day clinical work.

## Introduction

Patient portals, as an integral part of electronic health records (EHRs), are now available in nearly 90% of health systems in the United States, enhancing patient engagement and transforming patient–provider communication^1,2^. Through portal messaging, patients can contact their care teams outside of scheduled visits to ask questions, request medication refills, and follow up on lab-test results^3,4^. Incentivized by the Health Information Technology for Economic and Clinical Health Act^5^, messaging has become one of the most frequently used patient portal features^6^. Over the past decade, message volume has grown substantially^7,8^, with the COVID-19 pandemic further accelerating this surge, driving a 157% increase compared to pre-pandemic levels. This elevated rate of use has persisted since^9^. While enhanced communication is associated with improved patient care, it has also increased the burden on clinicians. The influx of messages has overwhelmed clinicians’ “in-baskets”—the EHR-based inboxes—resulting in a workload that often extends beyond regular work hours^8,10–12^. This sustained burden has been linked to clinician burnout, job dissatisfaction, and challenges in maintaining work–life balance^10,13–16^.

**Fig. 1:**
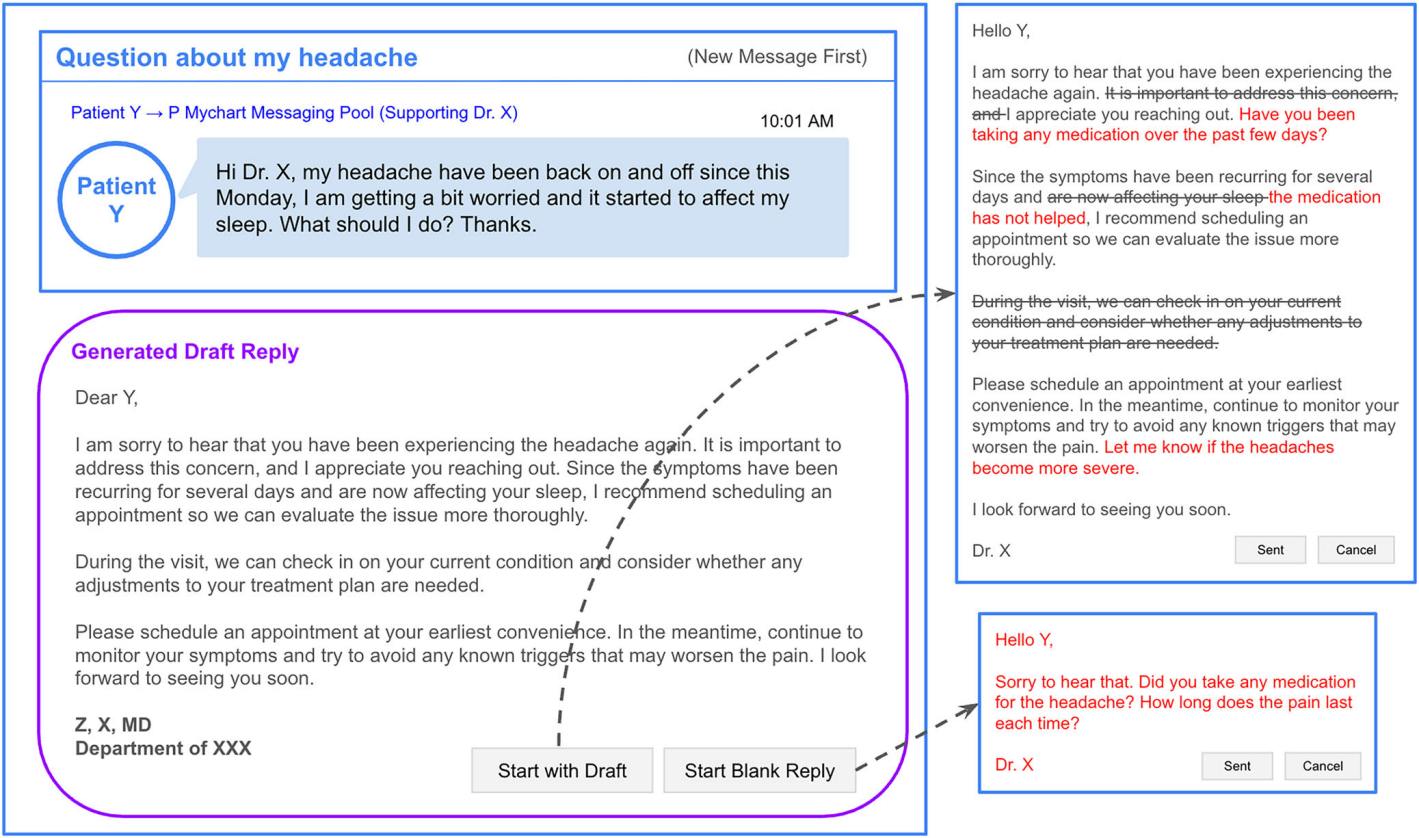
Workflow illustration for GenAI-assisted drafting of patient message replies.

Human, technological, and policy-level strategies have been adopted to address this growing burden. These efforts have included forming designated administrative teams^17^, refining management workflows^2,18^, developing artificial intelligence (AI) applications for message triage^19,20^, and introducing new billing codes for e-visits^21,22^. Most recently, generative AI (GenAI), particularly generative large language models (LLMs), has emerged as a potential solution for alleviating clinician in-basket overload^23^. Capable of interpreting complex texts and generating human-like responses, these models have demonstrated the ability to answer medical questions with expert-level knowledge^24,25^ and to respond to patient forum posts in a more empathetic tone than physicians^26,27^. With such capability, GenAI offers a novel approach to assist clinicians by creating draft replies to patient messages. Figure 1 illustrates how this technology works in general. The initial patient message is shown on the top left corner, and the AI-generated draft is shown below it. Clinicians can choose to compose the reply by using/modifying the AI-generated draft (top right) or by creating one from scratch (bottom right).

Several large health systems in the United States have begun implementing Health Insurance Portability and Accountability Act (HIPAA)-compliant GenAI tools for this purpose within their EHR systems^28–30^. These early adopters have highlighted GenAI’s potential to generate useful first drafts, reduce clinician exhaustion, and ultimately enable and improve the efficiency of asynchronous care^31,32^.

Despite encouraging progress, use of GenAI in facilitating clinical communication is still at an early stage, and the existing literature is highly fragmented. Prior reviews on GenAI in medical question-answering have mainly focused on its performance and efficacy in medical exams, clinical decision support, and patient education, rather than on its role in assisting clinicians with replying to patient messages^33–35^. Yet, when applied to emotionally engaged and potentially high-stakes patient–provider communication, GenAI may be associated with distinct ethical and operational concerns such as disclosure and patient consent regarding AI involvement in such interactions^36,37^. As interest in this application grows, there is no consensus to date on how to evaluate AI-drafted responses or what lessons can be drawn from initial explorations. Current studies vary widely in design, outcome measures, and evaluation methods. Clinical contexts for these studies also differ, spanning various specialties and diverse patient populations, each of which may present nuanced differences in communication practices. The lack of standardization presents challenges for synthesizing findings. These gaps underscore the need to assess the emerging body of evidence, both to guide the development and implementation of GenAI tools and to inform efforts to understand clinician and patient perspectives and preferences regarding AI-assisted communication. As these tools continue to evolve and become more integrated into care delivery, it is essential to ensure that they help reduce clinician burnout without eroding patients’ trust in healthcare providers and systems.

This review addresses these gaps by systematically identifying and synthesizing empirical studies that evaluate GenAI for drafting replies to patient messages through EHR-embedded patient portals. By examining study settings, objectives, designs, outcomes, and key findings, we aimed to provide a timely overview of the current evidence and outline directions for future research. Specifically, this review seeks to answer the following questions:

1. How have GenAI tools been studied for drafting responses to patient portal messages, and what are the study settings, objectives, and designs?
2. In what clinical contexts have these tools been evaluated?
3. Who are the users participating in these evaluations, and what approaches and outcome measures have been used?
4. What early consensus has emerged from the findings?

In answering these questions, we also discuss the challenges and opportunities surfaced by early studies and emphasize ethical considerations that should be prioritized to ensure the responsible use of GenAI in replying to patient messages. Our synthesis offers guidance for future evaluations and implementations of GenAI designed to aid in patient–provider communication, particularly through patient portals.

## Results

This section presents our synthesis of current research on the use of GenAI for drafting responses to patient messages. Table 1 summarizes the characteristics and quality assessment results of the included studies, and Table 2 provides an overview of the information extracted from these studies.

**Table 1:** Characteristics of included studies with MMAT quality assessment results

Study, year (article type other than full paper)	Study design	MMAT category of study designs	MMAT methodological quality criteria				
			1	2	3	4	5
Athavale et al.^54^, 2023	Cross-sectional evaluation study	Quantitative descriptive	Y	N	Y	Y	Y
Nov et al.^43^, 2023	Cross-sectional survey study	Quantitative descriptive	Y	Y	Y	Y	Y
Afshar et al.^38^, 2024 (Brief communications)	Pre-post quasi-experimental study	Quantitative non-randomized	CT	Y	Y	Y	Y
Baxter et al.^44^, 2024 (Perspective)	Retrospective qualitative evaluation study	Qualitative	Y	Y	Y	Y	Y
Chen et al.^55^, 2024 (Comment)	Two-stage observational study	Quantitative non-randomized	N	Y	Y	N	Y
English et al.^39^, 2024 (Research letter)	Prospective quality improvement study	Quantitative descriptive	Y	CT	Y	N	Y
Garcia et al.^31^, 2024	Prospective quality improvement study	Mixed methods	Y	Y	Y	Y	Y
Kim et al.^45^, 2024 (Research letter)	Cross-sectional survey study	Quantitative non-randomized	N	Y	Y	Y	Y
Liu et al.^46^, 2024	Model development and evaluation	Quantitative non-randomized	N	Y	Y	N	Y
Reynolds et al.^47^, 2024	Cross-sectional evaluation study	Quantitative non-randomized	N	Y	Y	N	Y
Robinson et al.^48^, 2024	Cross-sectional survey study	Quantitative non-randomized	N	Y	Y	N	Y
Scott et al.^49^, 2024	Cross-sectional evaluation study	Quantitative non-randomized	N	Y	Y	Y	Y
Small et al.^40^, 2024	Cross-sectional quality improvement study	Quantitative non-randomized	N	Y	Y	N	Y
Soroudi et al.^50^, 2024	Cross-sectional survey study	Quantitative non-randomized	N	Y	Y	Y	Y
Tai-Seale et al.^32^, 2024	Modified waiting list randomized quality improvement study	Quantitative randomized controlled trials	Y	Y	Y	N	Y
Tailor et al.^56^, 2024a	Cross-sectional evaluation study	Quantitative descriptive	Y	N	Y	Y	Y
Tailor et al.^57^, 2024b	Cross-sectional evaluation study	Quantitative non-randomized	CT	Y	Y	N	Y
Yan et al.^41^, 2024	Prospective quality improvement study	Quantitative non-randomized	N	Y	Y	N	Y
Cavalier et al.^58^, 2025	Survey-based factorial experiment study	Quantitative non-randomized	N	Y	Y	Y	Y
Hao et al.^51^, 2025	Retrospective observational study	Quantitative descriptive	Y	CT	Y	Y	Y
Hong et al.^42^, 2025	Framework development and evaluation	Quantitative non-randomized	N	Y	Y	N	Y
Kaur et al.^52^, 2025	Cross-sectional survey study	Quantitative non-randomized	CT	Y	Y	Y	Y
Tse et al.^53^, 2025 (Research letter)	Cross-sectional evaluation study	Quantitative descriptive	Y	N	Y	Y	Y

*Y* Yes, *N* No, *CT* Can’t tell.

**Table 2:** Summary of studies included in this review

Study	Setting and objective	Clinical context	Message and/or response	Evaluated responses by?	Participant	Outcome measure			Key finding
						Clinical expert	Patient/ Laypeople	Automated	
Athavale et al.^54^	Simulated O1	Vascular surgery	Devised 20 administrative non-complex and 20 complex medical questions on CVD based on actual messages via patient portal	GPT-4, GPT-3.5, Clinical Camel (a healthcare chatbot based on LLaMA)	1 internist and 1 vascular medicine specialist	Appropriateness, Completeness	N/A	N/A	ChatGPT-4 performed the best across both non-complex and complex question sets (100% and 75% appropriate and complete responses, respectively). ChatGPT3.5 ranked second for both sets. Reported one hallucination case from ChatGPT-3.5
Nov et al.^43^	Simulated, O3	Not specified	10 representative, non-administrative patient–provider interactions were extracted from EHRs	GPT-3.5, Human	392 layperson respondents from a US representative sample	N/A	Perception of authorship, Trust	N/A	On average, respondents correctly classified both AI and human responses around 65% of the time, with trust in chatbots being weakly positive but decreasing as task complexity increased.
Afshar et al.^38^	Live EHR, O2, O3, O4	Primary care, dermatology, oncology, psychiatry	AI drafts generated: 3882 (Pre); 3723 (Post); 2573 (Follow-up)	GPT-4 Human + GPT-4	Pre and post: 27 physicians Follow up: 44 with 17 nurses added	Thumbs up/down feedback	N/A	Utilization Edit distance	Total usage: 17.5%. Usage increased in the follow-up: 35.8%. Post prompt engineering, no change for utilization but decreased in “thumb down”. Only 2.6% AI drafts were used without or with minimal provider edits.
Baxter et al.^44^	Simulated, O1, O4, to evaluate whether LLMs can help address negative patient messages.	Not specified	A random sample of 50 negative sentiment messages and responses extracted from EHRs	GPT-3.5, Human	Two researcher coders. One is an ophthalmologist, and another has a master of public health and a doctoral degree	Themes in response	N/A	Length	AI responses were about triple the length of clinicians’. Differences were noted in relational connection, content, and next-step recommendations. Prompting mitigated some issues but not all. AI drafts could be helpful starting points but could escalate emotional conversations.
Chen et al.^55^	Simulated, O1, O2, O3	Oncology	100 hypothetical patient messages with simulated EHRs	GPT-4, Human, GPT-4 + Human	Stage 1 and 2 surveys: 6 attending radiation oncologists 2 additional physicians for content analysis	Helpfulness, Risk/Harm, Subjective efficiency, Content categories in response	N/A	Length	AI/AI-assisted responses were longer than human ones. About 7% of AI drafts posed a risk of severe harm or death. AI responses contained less direct action but provided more extensive education. Physicians reported improved efficiency, and responses became more consistent with AI assistance.
English et al.^39^	Live EHR, O1, O2, O3, O4	Primary care and specialty (non-specified)	AI drafts generated: 21323	GPT-4	12 nurses,14 MAs, 93 physicians and APPs	Empathy, Tone, Perceived efficiency, NPS, Minimal risk	N/A	Utilization	Overall, 12% utilization rate. Nurses were more likely to recommend the AI tool to others than MAs and clinicians, with more than 90% believing that it improved efficiency, empathy, and tone. Including the last A/P in prompts made some replies useful.
Garcia et al.^31^	Live EHR, O1, O2, O3	Primary care, Gastroenterology and hepatology	AI drafts generated: 9621	GPT-4	Total: 162 Primary care: 83 physicians and APPs, 4 nurses, 8 clinical pharmacists Gastroenterology and hepatology: 58 physicians and APPs, 10 nurses. Surveyed: 73	NASA TLX with a 4-item physician task load score derivative, PFI-WE score, NPS, Usability (utility, quality, perceived time saved), Free-text comment	N/A	Utilization, Change in reply action time, write time, or read time	Mean AI- draft utilization rate was 20%. No changes in reply action time, write time, or read time between pre-pilot and pilot periods. Task load and work exhaustion scores significantly decreased. Comments identified facilitators such as readiness, utility, and time-saving. Barriers included tone, content relevance, and accuracy.
Kim et al.^45^	Simulated, O1, O3, O4	Primary care, Endocrinology, Cardiology	59 messages selected from PMARs in EHRs	GPT-4, Stanford Health Care, and Stanford School of Medicine GPT, Human	6 clinicians 30 survey participants (layperson) recruited through the Stanford Research Registry	Empathy, Information quality	Satisfaction	Length	Promoted Stanford GPT was rated best for information quality and empathy. Satisfaction was higher with AI responses than with clinicians’ across specialties. Clinician responses were shorter. Satisfaction was not necessarily concordant with clinician-rated information quality and empathy. Clinician response length was associated with satisfaction while AI response length was not.
Liu et al.^46^	Simulated, O1, aimed to fine-tune a LLM using patient portal interactions as well as evaluate its responses	Primary care	Fine-tune: CLARE-Short (499286 portal message -response pairs) CLAIR-Long (the pairs + 5000 open-source patient questions with GPT-4 responses) Evaluate set: 10 representative, de-identified, and rephrased patient messages and responses	GPT-4, GPT-3.5, CLARE-Short, CLAIR-Long (based on LLaMA-65B), Human	4 primary care physicians	Accuracy, Empathy, Responsiveness, Usefulness, Free-text comment	N/A	BERTScore	GPT-4 responses were rated the best. ChatGPT models outperformed CLAIR-Long across accuracy, empathy, responsiveness, and usefulness. They all outperformed CLAIR-Short and the provider’ responses significantly. ChatGPT 3.5 achieved the highest BERTScore compared to actual provider responses.
Reynolds et al.^47^	Simulated, O1, O3	Dermatology	31 patient messages with questions related to dermatological conditions or management and their responses extracted from EHRs	GPT-3.5, Human	7 dermatology physicians, 3 nonphysicians	Overall quality‚ Readability‚ Accuracy‚ Thoroughness, Empathy, Preference	N/A	N/A	Both physicians and non-physicians preferred physician-generated responses over ChatGPT’s in most cases. Physician responses were rated significantly better in readability, empathy, accuracy, and overall quality. No hallucinations observed.
Robinson et al.^48^	Simulated, O1, O3	Urology	20 common BPH-related patient questions from phone or EHR-messaging were pooled, anonymized, and compiled	GPT-4, KPGPT (GPT-4-0613) SurgiChat (GPT-4-0613 with RAG on BPH literature), Human	2 urologists, 5 pre-screened non-medical volunteers that relevant to BPH	Accuracy, Empathy, Comprehensiveness	Perception of authorship, Preference, Empathy	N/A	Chatbot and urologist responses had similar accuracy, but chatbots rated significantly higher in completeness and empathy. Volunteers identified the correct author 59% and preferred chatbot responses. However, responses labeled as human scored higher in empathy than those labeled as chatbot.
Scott et al.^49^	Simulated, O1	Urology	100 patient messages requesting medical advice were collected from the in-basket of a urologist specializing in andrology.	GPT-3.5	5 urologists	Accuracy, Helpfulness, Completeness, Harmfulness, Intelligibleness, Acceptability	N/A	N/A	Overall, ChatGPT was rated to give accurate and intelligible answers, while completeness and helpfulness were rated lower. Harm was minimal. Performance was better on easier questions than harder ones. 47% of responses were considered acceptable.
Small et al.^40^	Live EHR O1, O2, O3	Primary care	117 unique HCP and 126 unique AI message-response pairs from pilot users’ in-basket AI drafts from silent validation, not being seen before)	GPT-4, Human	A convenience sample of 16 primary care physicians	Usability, Information content quality (completeness, accuracy, relevance) Communication quality (understandable, appropriate, tone; verbosity)	N/A	Length, Complexity (lexical diversity, Flesch-Kincaid grade level), Sentiment	Both AI and HCP responses were rated favorably. AI scored higher in communication style and matched HCPs in information content quality and usable draft proportion. Usable AI responses were seen as more empathetic, possibly due to their subjective and positive tone. They were also longer and more linguistically complex.
Soroudi et al.^50^	Simulated, O1, O2	Plastic surgery	10 queries from patients undergoing breast reconstruction with highest level of complexity and decision-making implications were extracted from EHRs	GPT-3, GPT-4, Human	2 APPs and 2 plastic surgeons for generated responses 2 medical students and 1 plastic surgeon, and 1 microsurgery fellow for review	Accuracy (surgeon and fellow) Empathy (medical students)	N/A	Length, FRE score, Time to compilation (self-reported)	Combined provider responses were more readable compared to combined chatbot responses. Empathy scores were higher in chatbot response. No significant differences in accuracy between providers and chatbot responses. Prompts increased readability.
Tai-Seale et al.^32^	Live EHR, O1, O2, O3	Primary care	10 679 replies to patient messages were examined	GPT-4	Immediate activation group: 25 physicians Delayed activation group: 27 Contemporary control group: 70	NPS	N/A	Length, Time spent reading and replying to messages	Access to AI drafts was associated with a significant increase in read time, no change in reply time, and significantly longer replies. Physicians’ views of AI replies ranged from helpful as starting drafts and for adding empathy, to ineffective, overly focused on visits, and having an overly nice tone. Examples showed physicians kept pleasantries from AI drafts but made substantive edits.
Tailor et al.^56^	Simulated, O1	Ophthalmology (across 9 subspecialties)	For each ophthalmic subspecialty, about 20 clinical questions were generated on the basis of common patient questions received via the clinic or patient portal	GPT-4	25 subspecialists participated. 22 of them both wrote and graded questions, and 3 only wrote questions.	Appropriateness	N/A	N/A	Reported robust aggregate appropriateness of an LLM across ophthalmic subspecialties both in the context of a patient information site (56%-100%) and as responses to EHR patient messages (54%-90%). Generally, inappropriate responses were inappropriate recommendations and incorrect or missing information.
Tailor et al.^57^	Simulated, O1, Q2	Ophthalmology	Created 21 retina questions similar to common patient inquiries received in clinic or via patient portals	GPT-3.5, GPT-4, Bard, Claude 2, Bing, Human, Human + GPT-4,	13 retinal specialists	Information quality Empathy, Safety (inappropriate/incorrect/missing content, extend/likelihood of possible harm)	N/A	Length, Time spent (self-reported)	For quality, Expert+AI performed the best overall while GPT-3.5 was the top performing AI. For empathy, GPT-3.5 got the best score followed by Expert+AI. There were time savings for an Expert+AI response versus expert-created response. ChatGPT-4 performed similarly to Expert for safety metrics.
Yan et al.^41^	Live EHR, O1, O2, O3, O4	Primary care	Train: 116 random PMARs and responses from 5 pilot physicians’ in-basket Validation: 200 additional PMARs and responses Test: AI drafts to 761 PMARs in production Patient validation 1&2: 250 PMARs and responses including the 116 in train set Patient validation 3: 250 PMARs and AI responses from production including the 200 in validation	GPT-4, Human	Test: 5 primary care physicians, 5 patient advisors Production: 69 primary care clinicians (physicians and APPs) including the 5 in test, the same patient advisors Post-production survey: 40 out of 69	Acceptance (Send, Edit or Reject), Helpfulness, Want to retain the tool? Recommend to colleagues? Perception of cognitive load reduced, Perception of time saved	Perception of authorship, Tone, Overall quality, Responsiveness	N/A	After prompt iterations, physician acceptance (send/edit) of AI drafts rose significantly, with 74% rated as helpful. Patients also reported improved tone and overall quality, noting most responses addressed patient questions. Patients were unable to distinguish between humans and AI for 76% of messages. Majority clinicians would like to keep the tool and would recommend it, 72% believe it can reduce cognitive load, and 41% believe it has potential to reduce in-basket time.
Cavalier et al.^58^	Simulated, O3	Not specified	Created 3 hypothetical patient messages representing low, medium, or high clinical seriousness 3 physicians wrote responses AI drafts were reviewed and minimally edited by two physician authors	GPT-3.5 + Human, Human	1455 respondents from patient advisory committee	N/A	Satisfaction, Perceived level of care, Usefulness, Preference for disclosure	N/A	Participants preferred AI responses over human responses regardless of the disclosure or seriousness of the topic. However, there was a slight decrease in satisfaction when told AI was involved. Participants preferred the shortest disclosure statement.
Hao et al.^51^	Simulated, O1, O2	Urology, radiation oncology	58 in-basket message interactions, selected from 90 patients with nonmetastatic prostate cancer	RadOnc-GPT (GPT-4 with RAG on local EHRs and oncology-specific database), Human	1 oncologist, 4 residents, 4 nurses	Completeness, Correctness, Clarity, Empathy, Estimated time to respond, Free-text comments	N/A	Inferences label, semantic similarity score, sentiment scores	RadOnc-GPT responses were more positive and generalized, while clinician replies had more balanced sentiment and greater variety. High similarity scores indicated strong content alignment. RadOnc-GPT slightly outperformed the care team in empathy, whereas it had comparable completeness, correctness, and clarity. Key limitations in RadOnc-GPT’s responses were lack of context, insufficient domain-specific knowledge, inability to perform meta-tasks, and hallucination. RadOnc-GPT was estimated to save clinicians time.
Hong et al.^42^	Live EHR, O1, O2, aimed to present a unified evaluation framework with mixed-methods metrics to assess AI for in-basket	Not specified	243 patient messages and AI draft pairs to establish and test human evaluation principles 42 patient messages from pilot users, with AI drafts, clinicians’ usage decisions, and final clinician-edited replies for compare qualitative and quantitative evaluations	GPT-4, Human + GPT-4	1 clinician reviewer	Relevance, Factuality, Completeness, Coherence, Clarity, Brevity, Toxicity	N/A	Perplexity, DiscoScore, FRE score, Compression ratio, Keyword Matching, ROUGE-N Recall|, BERT Toxicity	AI replies exhibit high fluency, clarity, and minimal toxicity, they face challenges with coherence and completeness. Most AI drafts were rated as usable with minor edits. However, reliability and accuracy of AI drafts are inconsistent across message categories. Clinicians’ manual decision to use AI drafts correlates strongly with quantitative metrics.
Kaur et al.^52^	Simulated, O1, O3	Primary care	20 unique patient message-response pairs from the medical center repository and de-identified 10 responses were generated by GPT, and the other 10 were written by real doctors	GPT-3.5, Human	8 primary care physicians for the initial review 49 HCPs for the evaluation	Accuracy, Readability, Empathy, Relevance, Perception of authorship	N/A	N/A	Compared with real doctors, GPT responses scored significantly higher in empathy and readability. However, no statistically significant difference was observed for relevance and accuracy. Participants correctly identified GPT messages 73% of the time and correctly identified authentic messages 50% of the time.
Tse et al.^53^	Simulated, O1	Primary care	Patient portal messages were randomly obtained from patients aged 12 to 17 years and their associated proxy users with EHRs (problem list, medications, and lab results)	GPT-4	Two pediatricians for review and a third pediatrician for resolving differences	Usefulness, Proxy user identification, Protection of confidentiality, Quality (relevance, factual correctness, literacy, conciseness, tone/style)	N/A	N/A	Among the proxy user messages, the AI correctly identified the proxy user 76% of the time. The AI disclosed unsolicited confidential information in fewer than 1% of cases. Most of the responses were in plain language, relevant, factually correct, concise, and 67% were rated as clinical useful.

*O1* evaluate the content of AI-generated drafts, *O2* evaluate the implementation of generative AI and its impact on clinical efficiency, *O3* understand user perceptions, preferences, or experiences, *O4* examine the effects of prompt engineering, *CVD* chronic venous disease, *MAs* medical assistants, *APPs* advanced practice providers, *NPS* net promoter score, *A/P* assessment and plan, *NASA TLX score* NASA task load index score, *PFI-WE* professional fulfillment index-work exhaustion score, *PMAR* patient medical advice request, *BPH* benign prostatic hyperplasia, *RAG* retrieval-augmented generation, *HCP* health care professional, *FRE* Flesch reading ease.

### Results of literature search and screening

**Fig. 2:**
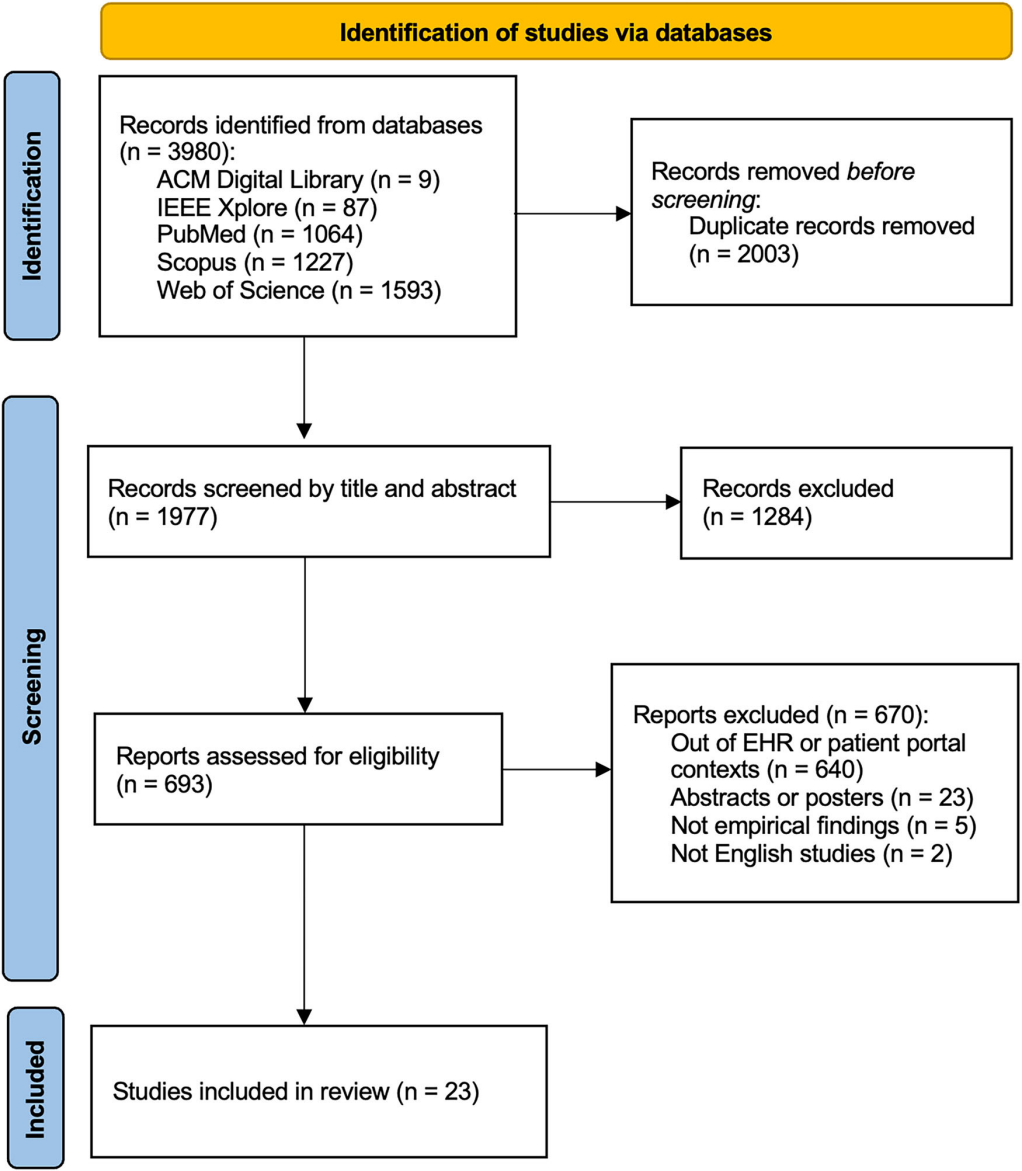
PRISMA flow diagram illustrating the study selection process.

Figure 2 shows the literature search and screening process following the PRISMA flow diagram format. Our search across five databases (ACM Digital Library, IEEE Xplore, PubMed, Scopus, and Web of Science) resulted in 3980 potentially relevant papers. After removing 2003 duplicates, 1977 articles remained. Screening based on title and abstract further excluded 1284 additional papers, leaving 693 for full-text review. After reviewing the full text of these 693 papers, 23 were deemed to meet the inclusion and exclusion criteria (detailed in the Methods section) and were included in the final review.

### Study characteristics

All 23 studies were conducted in the United States and published between 2023 and 2025. The majority of them (*n* = 16) appeared in medical and informatics journals, including *JAMA Network Open*, *Journal of the American Medical Informatics Association*, *JAMIA Open*, and *Mayo Clinic Proceedings: Digital Health*. The remainder (*n* = 7) were published in clinical specialty-focused venues, such as *Urology Practice*, *Ophthalmology Science*, and *Annals of Plastic Surgery*. In terms of type of publication, 17 were full-length research articles, while the others consisted of three research letters, one brief communication, one perspective, and one commentary.

### Study setting, objective, and design

The included studies evaluated GenAI for drafting replies to patient messages in two settings: live EHR systems (*n* = 7) and simulated environments (*n* = 16). Across these settings, studies primarily aimed to evaluate: (1) the content of AI-generated drafts (*n* = 19); (2) the implementation of GenAI and its impact on clinical efficiency (*n* = 8); (3) user perceptions, preferences, or experiences (*n* = 12); and (4) the effects of prompt engineering (*n* = 5). Many studies addressed more than one objective.

The live EHR system setting refers to real-world clinical environments where GenAI tools are embedded into existing in-basket workflows to generate draft replies for patient messages. All seven studies in this setting evaluated GenAI tools integrated into the Epic EHR (Epic Systems Corporation, Verona, Wisconsin, USA), using OpenAI’s Generative Pre-Trained Transformer (GPT)-4 for response generation^31,32,38–42^. Several studies explored GenAI implementation and its impact on clinical efficiency and user experience, using measures such as draft utilization^31,38,39^, extent of edits^38^, time spent^31,32^, and clinician burden^31,41^. Some evaluated user perceptions of AI-drafted content, aiming to understand how clinicians in different roles judged its usefulness^39^ or how linguistic features were associated with perceived empathy^40^. Three studies primarily examined prompt engineering. Two iteratively modified prompts in live settings and assessed their effect on draft usability and clinician feedback^38,39^. The third adopted a structured, human-in-the-loop process to refine prompts in a test environment before deploying them in production, evaluating their impact on provider acceptance and patient satisfaction^41^. A distinct effort sought to bridge qualitative and quantitative evaluation metrics by proposing a unified framework for assessing LLM performance in healthcare, which was then applied to Epic’s in-basket GenAI feature^42^. All implementations were conducted as pilot-scale efforts, with most framed as quality improvement projects^31,32,39–41^. The majority employed prospective^31,39,41^ or quasi-experimental^38^ designs to evaluate GenAI deployment or prompt modifications in real time. One study used a modified waitlist randomized design to compare outcomes between clinicians with and without access to GenAI drafts^32^, while another used a cross-sectional survey to capture provider perceptions of draft quality^40^.

In contrast, studies conducted in the simulated setting evaluated GenAI tools outside of live clinical workflows. These controlled experiments involved generating replies to either de-identified patient messages extracted from the EHR^43–53^ or hypothetical inquiries modeled on real patient portal communications^54–58^. The 16 studies conducted in this setting primarily focused on assessing the content quality^44–57^ and user perceptions^43,45,47,48,52,55,58^ of AI-drafted replies, using a range of outcome measures detailed later. Many compared GenAI drafts across different models^45,46,48,50,54,57^ or against human-authored responses^43–48,50–52,55,57,58^. Although not embedded in actual workflows, a few studies examined GenAI’s impact on efficiency through subjective ratings and self-reported time for responding or editing drafts^50,51,55,57^. While GPT-3.5 and GPT-4 were the most frequently used models, some studies evaluated alternative or customized variants. These included GPT-4-based, specialty-specific retrieval-augmented models^48,51^, fine-tuned versions of LLaMA adapted for clinical use^46,54^, and institution-developed GPT-powered tools^45,48^. One study also compared multiple commercial models, including Bard, Claude, and Bing, alongside GPT variants^57^. To approximate the EHR context, one study included simulated medical records alongside messages when prompting GPT^55^, while another connected a customized model to the local EHR system to access clinical notes and patient details in support of drafting^51^. A few studies also incorporated clinician editing processes to examine the human–AI synergistic effect in message drafting^55,57^ as well as to explore patient preferences regarding the disclosure of AI involvement^58^. Cross-sectional evaluations or surveys were commonly used to gather user perceptions and assessments, often through randomized and blinded review designs comparing responses by different GenAI models or humans^43,45,47,48,50,52,55,57,58^. Two studies had distinct objectives and approaches. One developed and fine-tuned a large language model on portal message data and evaluated its performance against baseline GPT and clinician drafts^46^. The other conducted a retrospective qualitative study focused specifically on GenAI responses to negative patient messages, using thematic analysis to compare content differences between GenAI and clinical care team replies^44^.

### Clinical context, message topic, and participant

GenAI for responding to patient messages was evaluated across various clinical contexts, with primary care, including internal medicine, family medicine, and pediatrics, being the most common setting (*n* = 10)^31,32,38–41,45,46,52,53^. Four studies conducted evaluations across primary care and specialties^31,38,39,45^, while the remaining studies focused on specific specialty domains, including dermatology^47^, urology^48,49,51^, ophthalmology^56,57^, oncology^55^, and surgery^50,54^.

Across the included studies, GenAI was used to generate draft replies for patient messages covering a variety of topics, ranging from administrative inquiries, such as appointment scheduling and medication refills^40,46,52,54,58^, to medical advice requests related to symptoms, postoperative concerns, and test results^49–51,56,58^. Several studies deliberately varied message seriousness^58^, complexity^54^, or level of detail^56^ to include comprehensive test cases, while others focused solely on clinical questions involving decision-making implications or condition-specific issues^41,43,45,47–51,56,57^. Some studies curated representative messages to ensure that evaluations covered the most commonly asked topics in patient portals^43,46,48,57^, while others randomly selected samples from the in-baskets of participating clinicians or from messages sent by a particular patient group^40,41,47,53^. To ensure fair GenAI evaluation under simulated conditions without integrated data sources, two studies excluded messages that required access to external information^40,46^. In addition, some studies collected unique message samples to meet specific research objectives. For example, one study evaluated GenAI using a mix of adolescent patient and proxy messages^53^, while another tested GenAI in emotionally sensitive scenarios using negative patient messages^44^.

Participants in the studies can be grouped into two high-level categories: clinical and non-clinical experts. Over 90% of the studies (*n* = 21) involved clinical experts across diverse contexts and roles, including physicians^31,38–41,44,46–50,52–57^, advanced practice providers^31,39,41,50,52^, nurses^31,38,39,51^, medical trainees (medical students, residents, and fellows)^50,51^, medical assistants^39^, and clinical pharmacists^31^. Clinician participants reviewed GenAI draft replies, shared pilot experiences, and either edited AI drafts or created human-authored counterparts for comparative analysis. In contrast, only a few studies engaged non-clinical participants, including patient advisors or laypeople^41,43,45,48,58^. These participants were typically asked to rate tone, identify the authorship, or share their personal preferences for the given responses, rather than evaluate their clinical quality. While considered laypersons in the medical context, many were active care stakeholders, such as long-term patient advisors with extensive experience using patient portals^41^, participants recruited from institutional research registries^45^, or pre-screened volunteers with a clinical condition of interest^48^. Only two studies focused exclusively on non-clinical perspectives. One surveyed over 1400 members of a patient advisory committee to evaluate preferences for AI-generated responses under varying disclosure conditions^58^. Another recruited a nationally representative sample of laypersons through a crowdsourcing platform to assess their ability to distinguish between human and AI responses as well as their trust in AI’s advice^43^.

### Evaluation method and outcome measure

All 23 studies incorporated human ratings using Likert scales to assess GenAI responses and their impact. Ten evaluations also calculated basic computational metrics, including text length, utilization rate, and time changes^31,32,38–40,44,45,50,55,57^, while only six studies employed more advanced computational metrics, such as BERTScore and Flesch-Kincaid grade level^38,40,42,46,50,51^. Among these six, one evaluation framework study intentionally mapped outcome measures to both human ratings and computational metrics to assess their alignment^42^. Building on the grouping approach used in prior work^40^, we categorized the common outcome measures examined in these studies into five groups: (1) information quality, (2) communication quality, (3) user perception, experience, and preference, (4) utilization and efficiency, and (5) composite measures.

Information Quality (*n* = 14). Information quality was consistently evaluated across the studies, encompassing measures such as accuracy^40,46–50,52^, completeness^40,42,48,49,51,54^, relevance^40,42,51–53^, and factuality^42,53^. These measures assess the integrity of the information presented in AI drafts, with accuracy (*n* = 7) being the most frequently evaluated. While similar or identical terms were often used, they may reflect subtle differences. For instance, some studies evaluated completeness by checking whether drafts lacked essential information needed to answer patients’ questions^40,42,49,51,54^, while others examined whether responses were comprehensive beyond the minimum required content^47,48^. Relevance or responsiveness was typically rated based on whether AI responses addressed patients’ concerns^40,41,46,52,53^. In contrast, studies using computational methods defined relevance by how well AI drafts inferred patient inquiries^51^ or matched clinician-authored replies^42^. Two studies treated information quality as a single dimension, without specifying the subcomponents it included^45,57^.

Communication Quality (*n* = 14). Communication quality was another important focus, capturing the patient-centered aspects of responses, with empathy (*n* = 9) being the most commonly measured aspect^39,45–48,50–52,57^. AI drafts were also evaluated for their tone or style, determining whether their wording and expressions were appropriate for the context of the conversation^39–41,53^. Readability^47,52^ and related subdimensions, such as clarity^42,51^, understandability^40,49^, and brevity (or verbosity)^40,42,53^, were assessed to ensure that responses could be easily comprehended by patients without semantic confusions, literacy challenges, or distractions. Several studies used computational metrics to assess these aspects, including DiscoScore, lexical diversity, and the Flesch reading ease score^40,42,50^. Two studies analyzed the overall sentiment of the replies^40,51^, while another calculated BERT Toxicity to detect any pejorative terms or non-inclusive language^42^.

User Perception, Experience, and Preference (*n* = 12). A variety of measures were used to evaluate user perceptions, experiences, and preferences regarding the use of GenAI to draft replies to patient messages. A recurring focus was the perception of authorship, specifically whether participants could distinguish between AI-generated and human-written replies^41,43,48,52^. Some studies also asked participants to indicate their preference or rate their satisfaction with each response^38,45,47,48,58^. Additionally, patients’ trust and perceived level of care from AI-generated replies were assessed^43,58^. GenAI’s impact on clinician burnout was examined in one study by comparing physician task load and work exhaustion scores in pre- and post-implementation surveys^31^. In parallel, a few other studies captured clinicians’ perceptions of reduced cognitive load, time savings, and improved efficiency to evaluate the potential benefits of GenAI assistance^39,41,55^. The net promoter score was also used as a measure of clinicians’ overall support for the GenAI tool^31,32,39,41^.

Utilization and Efficiency (*n* = 11). Measures related to utilization and efficiency often focused on basic characteristics or objective metrics of GenAI drafts and their implementation. Length (*n* = 7) was the most frequently evaluated characteristic^32,40,44,45,50,55,57^, as it may affect how efficiently clinicians review AI-generated drafts. Time-related measures included the time spent reading messages, as well as writing or editing replies. Some studies measured time changes before and after GenAI implementations^31,32^, while others compared the completion time of AI-generated, clinician-written, and clinician-edited replies^50,51,57^. Three implementation studies reported real-world utilization rates of AI drafts in clinical practices, typically measured by how often clinicians selected “Start with Draft” instead of “Start Blank Reply.”^31,38,39^ One of these studies also calculated the Damerau-Levenshtein distance between AI-generated drafts and final replies as a metric for estimating the editing effort required before clinical use^38^.

Composite Measures (*n* = 10). In several studies, some measures were framed to assess multiple dimensions of quality through subjective judgment. Appropriateness, treated as a composite concept, involves evaluating whether the tone of the message was suitable and whether the information provided was adequate in a reply^54,56^. Potential harm associated with AI-generated responses was assessed by considering not only the risk of incorrect content compromising patient safety but also the possibility of communication that appeared unfriendly or perpetuated bias^39,49,55,57^. Usefulness or acceptability was often rated based on whether clinicians believed that AI-generated responses could be directly sent to patients, used as a starting point, or help improve the quality of a final response^31,39–41,46,49^.

### Consensus from early findings

GenAI responses were generally found to match, and in some cases exceed, the quality of human-authored replies across several dimensions, though risks and limitations remain. GenAI drafts were frequently rated as comparable in information quality and more favorable in communication style compared to clinician-authored responses^40,45,46,48,50–52,57^. Empathy consistently emerged as a notable strength of GenAI drafts^39,40,45,46,48,50–52,57^, with GPT-4 most often recognized as the top-performing model^46,48,50,54^. However, potential harms associated with these responses, although minimal, were documented^39,42,44,49,51,53–57^. Common challenges included hallucinations, incoherent language, and limited contextual understanding^31,42,51,54,56^. One study found that 7% of AI-generated replies posed a risk of severe harm or death^55^, while another reported two instances in which AI disclosed unsolicited confidential information in messaging involving proxies^53^. Additionally, studies noted inconsistent AI performance across message types, with reduced reliability in clinically complex inquiries and an increased risk of escalating emotionally charged conversations^42–44,49,54,56^.

Laypersons—and even clinicians—often could not accurately distinguish between AI- and human-authored responses in blinded reviews. Relatedly, several studies asked participants to identify the authorship of responses and reported only low to modest accuracy, with averages ranging from 24% (among patients) to 73% (among clinicians), suggesting that AI-generated replies closely resemble human communication^41,43,48,52^. While several blinded evaluations showed that participants, particularly patients, tended to favor AI responses^45,48,58^, one study found that more empathetic and preferable responses were often attributed to human authorship, even when they were actually generated by AI^48^. Similarly, another study noted that disclosing AI involvement in reply drafting led to a slight decrease in patient satisfaction, although participants still valued transparency over nondisclosure^58^.

Despite positive attitudes, adoption of GenAI for drafting patient message replies in real-world settings remained limited. While recognizing GenAI’s limitations and the need for edits, clinicians still viewed these tools as helpful aids for managing inbox burden. Several studies highlighted that clinicians found the drafts acceptable or useful as starting points and appreciated features such as templates or pleasantries^32,40–42,44,46,47,49^. Many clinicians also expressed willingness to recommend GenAI tools to colleagues and to retain the tools in future workflows^31,32,39,41^. However, this enthusiasm did not translate into consistent use. Across pilot implementations, actual utilization of AI-generated drafts was low, with average usage rates no higher than 20%^31,38,39^. Few studies have explored the disconnect between perceived benefits and limited uptake.

While no strong evidence suggests time savings, current GenAI implementations were associated with perceived efficiency gains and burnout relief. Despite expectations for streamlining workflows, early implementations reported no statistically significant changes in message reply time^31,32^, and one study even observed an increase in read time following GenAI integration^32^. Moreover, all studies comparing length found that AI-generated responses were considerably longer than human-written ones, raising concerns about increased burden for draft review and editing^32,40,44,45,50,55,57^. Nevertheless, survey data revealed that clinicians perceived a meaningful reduction in task and cognitive load, along with decreased work exhaustion^31,41^. Some also reported a subjective sense of time saved and improved efficiency^31,39,41,55^, even in the absence of objective evidence for reduced time or workload.

Prompt engineering consistently emerged as an effective strategy for enhancing the quality and usability of GenAI drafts. Across studies, prompt optimization was associated with measurable improvements in response quality, tone, and user acceptance. One study reported a significant increase in clinician acceptance of AI-generated replies after three rounds of prompt refinement, alongside improvements in patient-rated tone and overall message quality^41^. Another found that a revised prompt led to a reduction in negative clinician feedback on drafts^38^. Incorporating the most recent assessment and plan into prompts was shown to improve perceived usefulness among clinicians^39^, while purposely designed prompts helped mitigate inconsistencies between AI- and clinician-authored responses, particularly in relational tone, content relevance, and clinical recommendations^44^.

## Discussion

The synthesis of current research demonstrates a growing interest among health systems in GenAI-assisted replying to patient messages, with various efforts to evaluate draft quality, impacts on clinical efficiency, user perceptions, and the role of prompt engineering. Although varied in study design, scope, and evaluation methods, these early explorations reached some consensus. GenAI-drafted replies were generally perceived as acceptable starting points, especially when enhanced by tailored prompts. Both clinicians and patients recognized its potential to alleviate in-basket burden and enhance patient–provider communication. However, current real-world adoption of GenAI drafts remains limited, and important concerns regarding performance reliability and potential patient risks persist. In this section, we examine the key methodological and implementation limitations, explore ethical considerations, and outline future directions for advancing the effective and responsible integration of GenAI into high-volume clinical in-basket workflows.

Early evaluations and implementations were often constrained in scope, scale, and generalizability. Most studies were conducted at a specific site, within a single health system, or involved relatively small sample sizes of message corpora and participants^32,39–44,48,51–53^. Several studies focused on clinicians from certain specialties or patient groups with limited demographic diversity, raising concerns about how well the findings translate across clinical settings or populations^31,43,47–50,54–58^. Evaluations were more commonly conducted in simulated environments rather than in live clinical workflows, with many studies assessing carefully selected single-turn messages or hypothetical inquiries^46,49–52,54–58^. As a result, these findings may not fully capture the complexity of real-world patient–provider messaging interactions, including diverse topics, contextual cues, or evolving patient conditions.

Studies employed diverse, often unvalidated evaluation rubrics and relied heavily on human judgment from evaluators with varying levels of clinical training^31,38,39,41,50–52^. Draft quality assessments were typically conducted by convenient samples of clinical experts^40,52,53^, and while most studies did not report inter-rater reliability, a few noted low to moderate levels of reviewer agreement^40,46,48^. Prompt engineering also differed widely across studies, with many relying on ad hoc or trial-and-error approaches, limiting the reproducibility of these optimizations^41,44,52^. Additionally, while poor rater agreement suggests diverse communication styles and preferences, current deployments fall short in supporting prompt personalization. This review also noted a lack of transparency around how GenAI tools integrated with EHR systems accessed and utilized medical records. Few studies reported details about incorporating problem lists, clinical notes, or messaging history in draft generation^31,51,53^, limiting the understanding of how GenAI contextually grounded their responses^52^. Without consistent access to comprehensive, up-to-date clinical information, the risk of generating inaccurate or context-agnostic replies increases.

Many early explorations acknowledged that patients were underrepresented^38,40,41,52,57^, with none of the current studies involving patients who actually received AI-generated replies during pilot implementations—pointing to a significant gap in which patient-facing impacts and their preferences were often inferred rather than directly assessed^45^. Another limitation in understanding user experiences is that, while a few studies collected and analyzed qualitative user feedback, efforts to explore user perspectives in depth remain absent. Without these explorations, it is difficult to interpret current facilitators and barriers^48,58^ or reconcile conflicting findings, such as disagreements among evaluators^40^ and the misalignment between positive perceptions and low real-world utilization. Differences in model performance and user perceptions across demographic subgroups were also largely unexplored^40,41,43,44,48^.

Several studies highlighted important ethical and legal considerations surrounding the responsible integration of GenAI into patient–provider messaging. Transparency—specifically whether and how to disclose AI contributions to patients—emerged as a key question to be addressed^39,43,48,58^. Despite findings that disclosure may reduce patient satisfaction^58^, upholding ethical norms in healthcare AI requires supporting patients’ right to be informed when AI is involved in the delivery of their medical information and care^39,58–60^. Concerns on biased model training, cultural insensitivity, lack of AI attribution, and unequal accessibility also raised important liability and equity implications^40,42–46,48,50,52,54,56,57^. However, current studies often flagged but rarely investigated these risks across patient subgroups. The findings underscore the need to align GenAI deployment with responsible principles from the outset across stages, addressing these issues proactively rather than reactively. Finally, in line with broader recognition in healthcare AI^61^, human oversight was consistently emphasized across studies as a key safeguard for ensuring the accountable and safe use of GenAI tools in clinical settings^42,44,48,50–53,55,57^.

Given the limitations and implications surfaced in early research, this emerging area presents substantial opportunities for advancement. Future research should build on the efforts of Hong et al.^42^ to continuously refine standardized evaluation frameworks that incorporate both human and computational assessments. A core set of validated and scalable measures would enable more reliable benchmarking, improve reproducibility, and inform best evaluation practices across settings and specialties. While the need for standardization is clear, it is also important to acknowledge the subjective nature of patient–provider communication. Future research should explore personalized GenAI prompting to accommodate individual variation and improve clinical relevance^31,32,40^. Moreover, as GenAI tools become more embedded in routine clinical workflows, the field would benefit from more longitudinal and multi-center trials to enhance the reliability and generalizability of outcomes^31,32,57^. Future work should include longer-term evaluations that track GenAI impact on response quality, clinician burnout, patient satisfaction, and clinical outcomes over time to better understand its real-world implications in care delivery. In parallel, greater attention is needed to examine how GenAI systems access and incorporate clinical information from the EHR^31,44,46^. Studies should also explore how this process might be customized by clinical specialty or patient group to improve draft relevance.

Patient perspectives should also be prioritized in future research^31,32,38,40,43,46,52^. While patients are the recipients of AI-generated replies, few studies have directly evaluated their experiences. The linguistic complexity of GenAI outputs may be manageable for clinicians but burdensome for patients with limited health or English literacy^40^. Future studies should assess patient expectations, comprehension, preferences, and satisfaction with GenAI-assisted replies and involve patients directly in the co-design of prompt engineering, usability evaluations, and disclosure or consent strategies. Additionally, a few studies reported that GenAI did not generate drafts for all patient messages during pilot deployments^32,41^. How the absence of certain drafts may lead to inconsistency in communication style and affect patient experience or trust warrants further investigation. Addressing fairness and mitigating model bias will also be essential to ensure GenAI systems function equitably across diverse patient populations^43,62^. To promote responsible GenAI applications, patient perspectives, along with ethical and inclusive considerations, should be prioritized and integrated early across all stages of design, development, evaluation, and implementation.

User training represents another critical area for future inquiry. Despite the recognized benefit of human–AI collaboration, clinicians currently work with GenAI drafts with limited guidance or support^31^. Targeted training programs are needed to help clinicians understand GenAI’s capabilities and limitations^56^, enabling them to make informed edits, prevent over-reliance, and ensure adequate oversight. At the same time, researchers, health system leaders, and policymakers should work to establish clear governance frameworks for AI-assisted messaging in response to ongoing calls for responsible AI in healthcare^63,64^. This includes addressing automation bias^43,46,55^, which can influence provider behavior, judgment, and decision-making, and ultimately affect patient outcomes. Additionally, the growing prevalence of billing for patient portal messaging may further complicate the integration of GenAI into patient communication^21^. This makes the need for guidelines and oversight even more pressing to safeguard patient satisfaction and trust. Institutions must develop clear policies on AI disclosure, informed consent, and data privacy to ensure transparency and uphold ethical standards in digitally mediated care.

This review contributes to the growing body of knowledge on using GenAI to respond to patient questions and inquiries, particularly within the EHR context, where it integrates medical records to assist patient–provider communication as part of clinical care. The synthesis presented here can be compared with existing reviews that examine GenAI as a tool for patient and medical education^25,34^, as well as literature investigating its use as a medical chatbot^65^. These comparisons help reveal the distinct benefits and challenges of GenAI applications across various health information-seeking settings and underscore the differences between viewing GenAI as a supportive assistant versus as an independent agent. Such distinctions may guide researchers and clinical stakeholders in further identifying appropriate directions and priorities. Additionally, as recent work has also begun to examine the use of LLMs to assist patients in writing efficient messages to their healthcare providers^66^, it is important to consider how AI’s presence on both sides of the communication may create interactive effects and reshape the patient–provider relationship.

This review processes several limitations. All studies included in this review were conducted in English and based in the United States, which limits the generalizability of our findings and does not reflect global efforts relevant to this topic. Generative AI applications in patient communication may differ substantially across countries due to variations in language, digital infrastructure, and regulatory environments. As such, this review may primarily inform contexts with similar health system characteristics. During the screening process, we also identified early studies that evaluated AI-generated replies using public patient inquiries from platforms such as social media or institutional websites, conducted prior to the availability of HIPAA-compliant tools^27^. While these studies provided useful early insights into the feasibility of GenAI-assisted messaging, we excluded them to maintain a focus on evaluations situated within health systems or EHR-integrated settings. We conducted a quality assessment using the MMAT; however, the quality results may be suboptimal due to the variability and exploratory nature of these early studies, which often aim to test feasibility and explore emerging practices rather than follow standardized methodological rigor. Despite these limitations, this review offers an important synthesis of empirical evaluations and highlights emerging opportunities and challenges in the integration of GenAI into patient–provider communication via portal messaging, addressing the growing interest in this rapidly evolving field.

In conclusion, this review systematically synthesizes early studies exploring the use of GenAI to draft responses to patient messages, highlighting the promising quality of AI-generated replies and the positive reception from both clinicians and patients. In addition to these encouraging findings, we also identified key limitations in the current evidence base, along with persistent risks and challenges to the effective and safe integration of GenAI into real-world clinical workflows. As these technologies continue to evolve, it is critical to establish shared evaluation standards, develop practical guidelines for disclosure and oversight, and engage diverse stakeholders in shaping responsible implementation. Our findings offer timely insights for health system researchers, leaders, and policymakers aiming to leverage GenAI as a novel tool to address clinician in-basket overload and enhance patient–provider communication via portal messaging.

## Methods

This review followed the 2020 Preferred Reporting Items for Systematic Reviews and Meta-Analyses (PRISMA)^67^ guideline for systematic reviews without meta-analysis to ensure methodological transparency and reproducibility. The review protocol was not registered in systematic review registries due to its focused scope and rapid timeline.

### Inclusion and exclusion criteria

This review focuses on original research articles that explore the use of GenAI to draft replies to patient messages within the EHR context. Eligible studies were required to present empirical findings from implementations, evaluations, or stakeholder perspectives. As this is an emerging area of research, we also included short reports such as brief communications, research letters, and perspective papers with empirical results. Studies were excluded if they were not published in English or focused solely on non-English outputs. We also excluded studies evaluating GenAI responses to general frequently asked patient questions on institutional websites or to inquiries posted on public platforms outside the EHR or patient portal setting. Patient-facing chatbots developed as standalone health assistants were also not considered. Lastly, we excluded non-peer-reviewed preprints, posters, and abstracts due to incomplete reporting, as well as non-empirical articles such as editorials, opinion pieces, and system design descriptions.

### Search strategy

A comprehensive literature search was performed on April 5, 2025, using five electronic databases: PubMed, Web of Science, Scopus, IEEE Xplore, and the ACM Digital Library. The search targeted metadata fields (title, abstract, and keywords) and was tailored to each database’s syntax. Search terms included “*generative artificial intelligence*”, “*patient*, *message*”, and “*response*” as well as their synonyms and variants. To maximize retrieval sensitivity, we also included the terms “*question*” and “*inquiry*”, which may be used in place of “*message*” in this context, to capture studies evaluating GenAI responses to patient messages described with different terminology. No time or language filters were applied during the search. A complete list of searching terms is provided in (Table 3).

**Table 3:** Literature searching strategy

**Generative AI terms**	“Generative artificial intelligence” OR “generative AI” OR “AI-generated” OR “AI generated” OR “AI-drafted” OR “AI drafted” OR “Artificial intelligence-generated” OR “artificial intelligence generated” OR “Artificial intelligence-drafted” OR “artificial intelligence drafted” OR “Large language model*” OR llm OR llms OR “Transformer model*” OR “pre-trained language model*” OR “generative pre-trained transformer*” OR chatgpt OR gpt
AND	
**Action terms**	Respond* OR response* OR reply* OR replies OR answer*
AND	
**Topic terms**	Patient* AND (messag* OR inquir* OR question*)

Table 3 lists the search terms and query logic used in the literature search of this review.

### Study screening and data extraction

Titles and abstracts were independently screened by two of four authors (DH, YG, YZ, LF). At this stage, without reviewing full-text content, we focused on identifying studies that explored GenAI for responding to messages, inquiries, or questions from patients. Disagreements were resolved through consensus discussions involving at least two authors. Articles meeting these criteria were retrieved for full-text screening. Each full text was independently reviewed by two of three authors (DH, YG, and YZ), based on the predefined inclusion and exclusion criteria. Any disagreements were resolved through discussion with the senior author (KZ).

Following the screening process, an initial data extraction template was developed based on the full-text review of the included studies and the research questions of this review. Two authors (DH and YG) performed data extraction for the first five studies and held meetings to adjust the template as needed. The final template captures study characteristics, context, objectives, design, participants, outcomes, findings, limitations, and implications. After finalizing the template, each included study was independently coded by the two authors. Discrepancies were discussed and resolved with input from the senior author (KZ), ensuring consistency and interpretive rigor across the dataset. Table 2 in the Results section reflects the key structure of our extraction template.

### Quality assessment

We conducted quality assessment using the Mixed Methods Appraisal Tool (MMAT)^68^, a critical appraisal tool developed for systematic mixed-studies reviews. The MMAT provides evaluation criteria across five study types: qualitative research, randomized controlled trials, nonrandomized studies, quantitative descriptive studies, and mixed methods. These categories reflected the study designs included in our review. Two authors (DH and either YG or YZ) performed the assessments independently and resolved any disagreements through discussion. Ratings for each criterion were based on informed judgment guided by the MMAT manual, considering the study objectives, context, and the developmental stage of research in this area. As encouraged by the MMAT, we reported criterion-level ratings in (Table 1).

## Supplementary information

Supplementary information

## Data Availability

Data is provided within the manuscript or supplementary information files.
